# Mobile Apps for Medication Management: Review and Analysis

**DOI:** 10.2196/13608

**Published:** 2019-09-11

**Authors:** Katarina Tabi, Abnashi Singh Randhawa, Fiona Choi, Zamina Mithani, Friederike Albers, Maren Schnieder, Mohammadali Nikoo, Daniel Vigo, Kerry Jang, Regina Demlova, Michael Krausz

**Affiliations:** 1 Department of Psychiatry University of British Columbia Vancouver, BC Canada; 2 Department of Pharmacology Masaryk University Brno Czech Republic; 3 Centre for Health Evaluation and Outcome Sciences St. Paul’s Hospital Vancouver, BC Canada; 4 Center of Human Movement Sciences University of Groningen Groningen Netherlands; 5 Wolfson School of Mechanical, Electrical and Manufacturing Engineering Loughborough University Loughborough United Kingdom; 6 Faculty of Health Sciences Simon Fraser University Vancouver, BC Canada; 7 Department of Global Health and Social Medicine Harvard Medical School Boston, MA United States

**Keywords:** eHealth, mHealth, drugs, pharmaceuticals, therapy

## Abstract

**Background:**

Pharmacotherapy remains one of the major interventional strategies in medicine. However, patients from all age groups and conditions face challenges when taking medications, such as integrating them into the daily routine, understanding their effects and side effects, and monitoring outcomes. In this context, a reliable medication management tool adaptable to the patient’s needs becomes critical. As most people have a mobile phone, mobile apps offer a platform for such a personalized support tool available on the go.

**Objective:**

This study aimed to provide an overview of available mobile apps, focusing on those that help patients understand and take their medications. We reviewed the existing apps and provided suggestions for future development based on the concept *understand and manage*, instead of the conventional *adhere to medication*. This concept aims to engage and empower patients to be in charge of their health, as well as see medication as part of a broader clinical approach, working simultaneously with other types of interventions or lifestyle changes, to achieve optimal outcomes.

**Methods:**

We performed a Web search in the iOS Apple App Store and Android Google Play Store, using 4 search terms: medication management, pill reminder, medication health monitor, and medication helper. We extracted information from the app store descriptions for each eligible app and categorized into the following characteristics: features, author affiliation, specialty, user interface, cost, and user rating. In addition, we conducted Google searches to obtain more information about the author affiliation.

**Results:**

A total of 328 apps (175 Android and 153 iOS) were categorized. The majority of the apps were developed by the software industry (73%, 11/15), a minority of them were codeveloped by health care professionals (15%, 3/20) or academia (2.1%; 7/328). The most prevalent specialty was diabetes (23 apps). Only 7 apps focused on mental health, but their content was highly comprehensive in terms of features and had the highest prevalence of the education component. The most prevalent features were reminder, symptom tracker, and ability to share data with a family member or doctor. In addition, we highlighted the features considered innovative and listed practical suggestions for future development and innovations.

**Conclusions:**

We identified detailed characteristics of the existing apps, with the aim of informing future app development. Ultimately, the goal was to provide users with effective mobile health solutions, which can be expected to improve their engagement in the treatment process and long-term well-being. This study also highlighted the need for improved standards for reporting on app stores. Furthermore, it underlined the need for a platform to offer health app users an ongoing evaluation of apps by health professionals in addition to other users and to provide them with tools to easily select an appropriate and trustworthy app.

## Introduction

With the population aging worldwide [1] and increasing lifespans, the rates of chronic health conditions are accelerating [2]. Consequently, the regular use of medication to manage these conditions is increasing [3,4]. Moreover, there is also an increase in the number of individuals taking several medications simultaneously, known as polypharmacy [5,6]. The US National Health Survey has reported that the prevalence of polypharmacy (ie, taking ≥5 medications) rose from 8.2% to 15% between 1999 and 2012 [5]. This increase was found in all age groups, with the greatest increase among young adults aged 20 to 39 years, in which polypharmacy had grown from 0.7% to 3.1% [5].

Taking several medications simultaneously or starting to take a medication that is prescribed for the first time may bring with it challenges for many users [7,8]. These challenges may include integrating the medication into the daily routine at the right time, understanding the medication and its effects and side effects [9], or dealing with concerns about medication safety [10] and efficacy. Some patients want to organize their medications, keep a history or a list of currently used medications readily available, or track their symptoms in relation to the treatment [11]. To achieve these goals, a reliable and easy-to-use medication management (MM) tool that facilitates patient engagement becomes critical. The literature confirms that the patient’s compliance with the treatment is significantly improved when the patient is engaged in the treatment [12].

Mobile apps can offer users (patients or health consumers) tools to facilitate their engagement in the treatment and well-being [13,14]. In addition, mobile apps represent one of the solutions for challenges with MM [15] simply because 95% of US adults in 2018 owned a cellphone [16,17], and it allows MM solutions to become highly personalized. Moreover, people carry cellphones with them for the majority of the day, so MM apps can provide assistance *on the go* and in the required time [16,18].

App users as well as developers are realizing this potential, which is reflected in the mobile app market growth, with increasing numbers of MM apps available [15]. However, there are a limited number of studies that provide detailed characteristics of the currently available solutions [15]. The term electronic health (eHealth) solution is a general term that refers to software, telecommunication, and virtual technology related to health [19]. Some of the earlier studies focused on apps with selected features, such as reminders and other medication adherence strategies [20-22], or on a specific population [23]. Therefore, to inform further app development, it is crucial to explore the existing selection of solutions available in the market, review their key characteristics, and identify the needs and directions for future development.

This study aimed to provide an overview of the existing solutions in the mobile app marketplace, with a focus on apps that help users understand and take their medications. We reviewed the existing apps and provided suggestions for future development based on the concept *understand and manage*, as opposed to the conventional *adherence to medication*. This concept aims to engage users and empower them to be in charge of their health and well-being as well as to see medication as part of a broader clinical approach, working simultaneously with other types of interventions or lifestyle changes, to achieve optimal outcomes.

We outlined the detailed characteristics of the available apps and sought to (1) identify who designed each app and whether health experts were involved in the development and design; (2) identify key features defined as (a) features that are most prevalent and (b) features that are novel or innovative and have the potential to address the concept *understand and manage*; and (3) create a framework to categorize the mobile health solutions. We believe that this framework may contribute to the understanding of the current landscape of MM apps and to the development of future solutions.

## Methods

### Overview

The approach used in this study was based on the previous literature [15,24,25]. The app search was conducted on the 2 main mobile app stores: Android Google Play Store (from here on referred to as Android store) and iOS Apple App Store (from here on referred to as Apple store). Worldwide mobile phones using these 2 operating systems (ie, iOS and Android) currently account for 96% of the mobile operating system market share, and in North America and Europe, they account for 99% of the market share [26]; the market share numbers for newly purchased devices are even higher [27]. The app stores were searched in January 2017. The search query was typed into the search engine Start page (details described in the section *Eliminating Personalization* *During the Search*). The query consisted of the search term and the app store, for example, *medication management site://play.google.com/store/apps/*. The search terms used were *medication management, pill reminder, medication health monitor,* and *medication helper*. The selection of search terms was based on a panel discussion among the research team and terms used in previous studies. The final search terms were confirmed based on a preliminary search, when the selected terms showed greater relevance of results. Table 1 lists the search terms together with the number of apps found with them in each app store.

**Table 1:** Search terms and the number of eligible apps found with them in each app store.

Search term	Number of apps found that met the inclusion criteria	
	Apple store	Android store
Medication health monitor	69	70
Medication management	53	61
Pill reminder	49	94
Medication helper	53	54

### Selection of Eligible Solutions

Each term was searched separately in each app store, and 2 researchers performed identical searches independently. For all search results, the information available in the app store description was reviewed. The inclusion criteria were as follows: (1) user population consisting of patient and health consumers, including family or caregivers, and the eligible solution was written in lay language; (2) purpose—helping users take or understand their medication; and (3) the app was available in English. Apps were excluded from further review if professional language was used, they were intended for health care experts, and they did not have a clear focus on helping take medications and instead focused on other issues, such as purchasing medications or ordering refills.

Solutions that met the eligibility criteria were selected by the researcher and their URLs were saved. After the independent search, the 2 researchers compared the lists of apps they identified as eligible and performed analyses to determine agreement between the 2 sets of results. Interrater reliability was determined based on Cohen kappa (0.81). In accordance with Landis and Koch statistics guidelines, the strength of the agreement between researchers was almost perfect (0.81-1.00).

Only those apps that were considered ineligible by both researchers were excluded from further review. Discrepancies between the 2 researchers were reviewed (third opinion) and resolved. Subsequently, both researchers agreed on the final list of results for each search term [15].

Duplicates on the same device platform were identified using Microsoft Excel and removed, whereas solutions that appeared across 2 different platforms were not considered duplicates [28]. Subsequently, the final list of unique solutions meeting the eligibility criteria was created.

### Eliminating Personalization During the Search

We applied the following strategies to decrease the extent to which the search results were affected by personalization, app store search algorithm, and cookies. We used (1) Start page as the search engine, which uses results from Google while offering increased anonymity through Secure Sockets Layer encryption and limited data collection; (2) a different Web browser than is usually used on the computer; and (3) incognito mode or a private window of the browser [29,30].

### Data Extraction and Solution Assessment

Before data extraction, a preliminary framework of app characteristics was designed, based on a panel discussion of the research team and the previous literature [15,24,31]. Later, the framework was shaped according to information that accumulated during the selection of eligible solutions. The final framework of the solution characteristics is outlined in Table 2.

**Table 2:** Final framework for app characteristics categorization, including description for selected subcategories.

Categories of app characteristics and subcategories		Description
**Feature or purpose**		
	Medication reminder	Reminds user to take medication dose in real time
	Shares data and reports with others	When chosen, ability to share health data managed in the app with other people (family member and health care professional)
	Education about medication	Information about medication (eg, benefits, side effects, interactions, and use)
	Identifies pills	Identifies unknown pill, usually through the phone’s camera and the appearance
	Checks for drug interactions	After entering two or more medications (or medication with food or alcohol), the checker will evaluate the risk of their interactions
	Tracks symptoms, side effects, health data, and vitals	Tracks user’s measurements (eg, blood glucose, blood pressure, weight, pulse, temperature, mood, and sleep patterns), symptoms of disease, and side effects
	Manages profiles of multiple users	Within one app, there is an option to have medication profiles of several people (eg, other family members)
	Synchronization with other apps or devices	The app has the ability to synchronize itself and the entered data with another mobile app or device (eg, Apple Watch)
	Data privacy and security	Different forms of information privacy (eg, password) and security; privacy policies are transparent and easy to find; and advanced protection of health data
	Medication management (MM) is not the primary aim of the app	Medication-related functions are not the primary focus of the app, for example, many fitness apps have mainly other features, and the medication component simply represents a minor piece
	Other features	Describing any other features, not listed above
**Author Affiliation**	
	
	Academia	Developed by or in affiliation with university or other forms of academia
	Health care professionals	Developed by or in affiliation with health care professionals, health centers, and hospitals
	Software industry	Including software companies or independent software developers
	Other	Affiliations not listed above; usually charities, nonprofits, and government organizations; pharmaceutical companies and insurance companies; various level of health care professional involvement; therefore, the apps under this subcategory may have input from health experts
	Insufficient information	The affiliation cannot be found
**Specialty in Medication**	
	Diabetes	App specialized in MM for diabetes-specific medication
	Women’s reproductive health	App specialized in the management of contraceptive pills or women’s reproductive health-specific medication
	Cardiovascular health	App specialized in MM for heart and blood circulation–specific medication
	Lifestyle management	App specialized in lifestyle management, often offering MM as one of the several features
	Neurology	App specialized in MM for neurology-specific medication
	Mental health	App specialized in MM for mental health–specific medication
	Oncology	App specialized in MM for cancer-specific medication
	Hematology	App specialized in MM for hematology-specific medication
	Lungs, allergy, and immunity	App specialized in MM of drugs for allergy, asthma, lung disease, and immune system–specific medication
	Veterinary medicine	App specialized in MM for pets
	Digestive system	App specialized in MM for gastrointestinal-specific medication
	Other specialty	Specialty not included in the list
	Without specialty	General MM, for use with any medication
**User Interface**	
	Static only	Provides minimal interaction with the user—does not work with individual user’s data, just displays the same content for everybody; only available interaction is for navigation and settings; usually presents information
	Single dynamic feature	Provides one interactive component; compared with the multiple dynamic category, apps in this category often have only a basic pill reminder and no other features
	Multiple dynamic features	Solutions in this category are more comprehensive, with more than one dynamic feature. Dynamic user interface is defined as providing the opportunity to input one’s individual data into the app and being able to interact with the app (eg, tracking health data, warning if set parameters are exceeded, games, and communication)
**Cost**	
	Free	The app itself was available for free. However, some of the apps in this category had the option of purchasing extra content or features (referred to as in-app purchases). These apps were coded as free and the cost for the optional in-app purchases was listed at the same time—under the next column cost
	Price	Includes price for the app itself or price for the in-app purchases (available also in free apps)
	Currency or country	Encoded to determine the currency that was used; later, all the prices can be converted into the same currency for comparison and descriptive statistics
	Notes	Explains details related to the listed price—whether the price is for the app itself, regular subscriptions, or in-app purchases
**User Rating**	
	Number of stars	1 star-5 stars
	Number of ratings per app	Total number of ratings in the app store available for the given app; if the information was presented by the app store

Subsequently, the 2 researchers assessed and categorized the apps. The first 20.1% (66/328) of the apps were categorized by the 2 researchers together. During this process, they came to an agreement in understanding the scope of the subcategories. Subsequently, they divided the remaining sample of apps (262) and each researcher extracted information for half of the sample. They assessed the apps at the same time and discussed any issue or uncertainty that arose.

Information was extracted from the app store description and from the available screenshots. An exception was the category author affiliation. The developers rarely described their credentials or affiliation within the text of the app store description. Moreover, app stores provided only the name of the developer in most cases. Therefore, to find out more information about the author’s affiliation, we conducted Google searches using the name and the website provided by the app store.

The app features were assessed in the following way. Features that were not part of the list of prevalent features were manually entered in the database as *Other features*. At the same time, researchers flagged those features, which they considered novel, interesting, and innovative. Upon completion of the solution assessment, the team of coauthors reviewed the flagged features and agreed on the final list, which is included in the study as the *novel and innovative features*.

### Data Analysis

Once all the apps were coded, descriptive statistics were computed for each variable. Cohen kappa and selected descriptive statistics were computed using SPSS (IBM), version 25. The rest of the descriptive statistics and charts were prepared in Microsoft Excel, version 16.1.1 and Euler online application. The visualization through Euler diagram and related descriptive statistics were presented to add to the understanding of who authored the apps and which professionals collaborated during the app development.

## Results

### Search and Categorization

**Figure 1:**
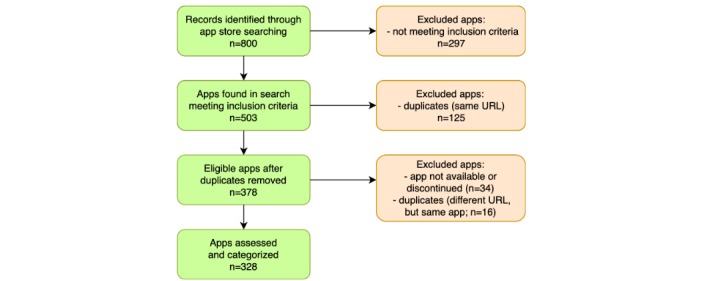
Diagram illustrating the review process and exclusion of apps at various stages of the study.

The initial app store search yielded 800 records, of which 297 were excluded based on the app store description because they did not meet the inclusion criteria. From the eligible apps, other apps were excluded later because they were duplicates or they were no longer available. A total of 328 apps were included in the final assessment. Categorization was carried out in December 2017. Between the search and categorization, 34 apps were discontinued. Therefore, the apps that were assessed and are the subject of this review are the ones that remained available on the app stores for a year. Figure 1 demonstrates the various stages of the review process.

### General Characteristics of Included Apps

Both marketplaces were represented in the final list of apps: the Android store accounted for 53.8% (175/328) of the apps, and 46.6% (153/328) of the apps were from the Apple store. In terms of cost, the majority of the apps were available for free (86.3%; 283/328), some of which had options for in-app purchases (9.5%; 31/328) for extra content or features. The average price of paid apps (13.7%; 45/328) was US $3.77. The price range for the paid apps or in-app purchases was between US $0.99 and $24.55.

### Authors and Affiliations

**Figure 2:**
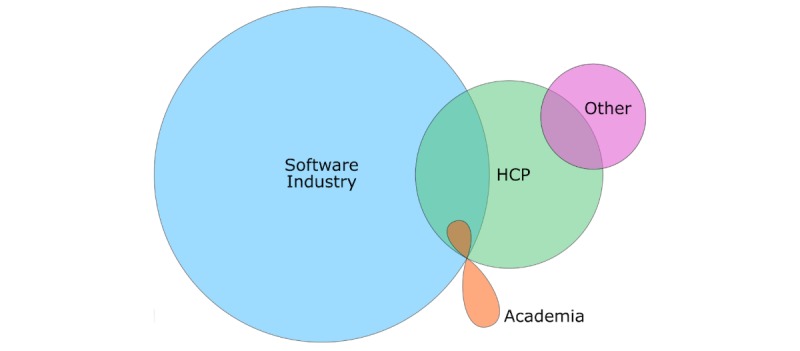
Diagram illustrating the distribution of different authors involved in the development of the medication management apps. The overlapping regions indicate the apps codeveloped by 2 or 3 different author affiliations (combinations). HCP: health care professionals.

The majority of the mobile apps were developed by the software industry (72.9%; 239/328), which consisted of software companies (219/239) and independent software developers (20/239). In addition, 14.6% (48/328) were developed with the involvement of health care professionals. Very few apps were developed by, or produced in collaboration with, academic institutions (2.1%; 7/328). Furthermore, 5.2% (17/328) were developed by *Others*, which consisted mostly of governmental organizations and nonprofits; for a longer list of institutions, see Table 2. In 56 cases (17.1%; 56/328), there was insufficient information available about author affiliation. The distribution of different authors or developers, including the combinations, is illustrated in Figure 2.

### Features

The most prevalent features of all reviewed apps are listed in Table 3. From the rest of the features, those identified as novel or innovative, are listed in Table 4. From the apps included in the study, 77 apps did not have MM as their main purpose. The main focus of these apps was usually overall health management, and a feature related to medication was not one of their primary features. Some of these apps were supporting healthy life style choices and a broader clinical approach to health, including tracking physical activity, food intake, sleep, emotions, and others.

**Table 3:** The most prevalent features of the mobile apps for medication management.

Features	Number of apps that have the feature
Medication reminder	282
Tracks symptoms, side effects, health data, and vitals	152
Shares data and reports with others	135
Synchronization with other apps or devices	70
Education	63
Manages multiple user profiles	60
Data privacy and security	45
Checks for drug interactions	12
Identifies pills	11
Medication management is not the primary aim of the app	77

**Table 4:** Novel or innovative features of the apps.

Feature description	Examples
Novel ways of data entry, instead of typing	Scanning barcode, taking a picture of the package, and voice entry
Correlations based on entered data	Medication-blood pressure, food-glucose, and medication-sleep
Communication system with the health care professionals	Connecting user with pharmacist, doctor, nurse, or other professionals
Goal setting	For physical activity, diet, sleep hygiene, social interactions, and other lifestyle choices
Journaling	Accomplishments, emotions, triggers, pain, sex drive, mood and thought journal, and other
Reminder noticing the change of time zone	Support during traveling
Reminder with personalized voice	Voice of grandchild reminds the grandmother
Warning if safe dosage exceeded	App includes information about the recommended maximum daily dosage and warns if that is exceeded
uBox (place where the medication can be physically stored) synchronized with the app	Option to set: care contacts receive text message when patient misses a dose
Safety plan for acute situations	Prepared ahead when the health condition is stable or prepared together with the health care professional
Emergency button	911 and counselor

### Specialty in Medicine

**Figure 3:**
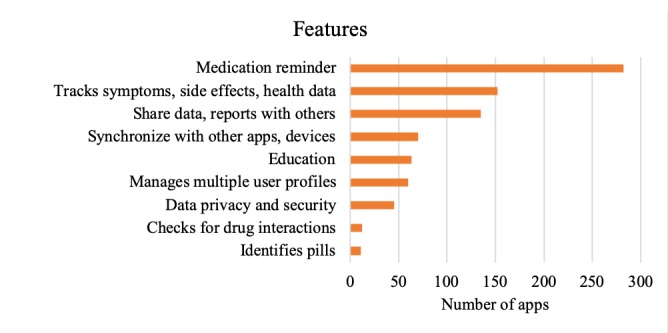
Distribution of medication management (MM) apps focusing on certain medical conditions or specialties in medicine.

Of the apps focusing on certain medical condition and its specific MM (36.0%; 118/328), most apps focused on diabetes (n=23), women’s reproductive health (n=20), and cardiovascular health (n=15; see Figure 3). Mental health was not 1 of the 3 most prevalent specialties. Of the 7 apps focusing on MM for mental health, 5 were from the Apple store, whereas only 2 apps were found in the Android store. Most apps included general support for mental health treatment; 2 apps were more specialized, focusing on attention-deficit/hyperactivity disorder and bipolar disorder.

Overall, 210 apps did not specify a specialty (64.0%; 210/328), which means they were designed to help with general MM. These general MM apps may differ from the specialized apps by nature of their features, which are designed to address a wide spectrum of medications and health conditions. For example, the tracker of health data in a general MM app usually offers tracking of weight, heart rate, sleep, or exercise. Conversely, a specialized MM app for mental health may track some of the previously listed health data in addition to mood, thoughts, and social events. Some users may prefer the general MM apps. For example, a user taking several medications across a range of specialties can manage all their medications within one app and customize the settings accordingly.

Another way of looking at the apps’ features is to see the relationship between features and medical specialty. Table 5 shows what features were prevalent in apps for diabetes, cardiovascular health, and so on and compares them with other specialties. For example, the table shows that 61% (14/23) of the diabetes apps had reminders, 57% (13/23) of them had the option to share data with others, 22% (5/23) had an education component, 4% (1/23) could check interactions, 91% (21/23) tracked symptoms and vitals, and so forth. Specifically, Table 5 shows that the diabetes apps had the greatest variety of features (9 different features are represented). In contrast, the veterinary apps had the lowest variety, with only 3 different features. Not surprisingly, mental health MM apps had the highest prevalence (43%; 3/7) of the education component.

**Table 5:** Distribution of medication management apps by feature and specialty in medicine.

Specialty	Number of apps having the given feature within the specialty, n1 (%)^a^									Number of all apps within the specialty, n2
	Rem^b^	Sha^c^	Edu^d^	Int^e^	Tra^f^	Mul^g^	Syn^h^	Not^i^	Sec^j^	
Diabetes	14 (61)	13 (57)	5 (22)	1 (4)	21 (91)	4 (17)	8 (35)	16 (70)	1 (4)	23
Women’s health	18 (90)	1 (5)	2 (10)	0 (0)	9 (45)	0 (0)	3 (15)	3 (15)	5 (25)	20
Cardiovascular health	12 (80)	6 (40)	1 (7)	0 (0)	12 (80)	2 (13)	6 (40)	7 (47)	1 (7)	15
Lifestyle management	11 (100)	7 (64)	1 (9)	0 (0)	11 (100)	1 (9)	6 (55)	7 (64)	1 (9)	11
Neurology	6 (86)	4 (57)	0 (0)	0 (0)	7 (100)	0 (0)	5 (71)	5 (71)	0 (0)	7
Mental health	6 (86)	5 (71)	3 (43)	0 (0)	6 (86)	0 (0)	1 (14)	4 (57)	2 (29)	7
Oncology	4 (80)	2 (40)	2 (40)	0 (0)	4 (80)	0 (0)	1 (20)	2 (40)	0 (0)	5
Hematology	3 (75)	3 (75)	0 (0)	0 (0)	4 (100)	0 (0)	2 (50)	2 (50)	1 (24)	4
Allergy, lungs	3 (75)	3 (75)	0 (0)	0 (0)	3 (75)	2 (50)	1 (25)	2 (50)	0 (0)	4
Veterinary—medication management for pets	4 (100)	0 (0)	0 (0)	0 (0)	2 (50)	1 (25)	0 (0)	0 (0)	0 (0)	4
Digestive system	1 (33)	0 (0)	0 (0)	0 (0)	2 (67)	0 (0)	1 (33)	2 (67)	0 (0)	3
Other specialty	11 (73)	7 (47)	4 (27)	0 (0)	7 (47)	0 (0)	4 (27)	10 (67)	0 (0)	15

^a^Percentage (%) calculated as (n1/n2) × 100.

^b^Rem: Reminder.

^c^Sha: Share data.

^d^Edu: Education and information.

^e^Int: Interactions.

^f^Tra: Tracks symptoms and health data.

^g^Mul: Multiple profiles management.

^h^Syn: Sync with other apps or devices.

^i^Not: Medication management not primary aim.

^j^Sec: Data security.

### User Interface

Apps with only a static user interface accounted for 0.6% (2/328) of the sample. A definition of different user interfaces is provided in Table 2. The remaining apps had a dynamic user interface. We defined this as providing an opportunity to input one’s individual data into the app (medication type, use times, tracking health outcomes, or other individual information), and the possibility to interact with the feature. Of the dynamic apps, 99 (30.2%; 99/328) had only 1 dynamic feature (usually a basic pill reminder), whereas 227 (69.2%; 227/328) apps had multiple dynamic features available, and the complexity of these apps was higher.

### Rating

The user rating scale was slightly different for the Android and Apple stores. The Android store showed ratings with an accuracy of 0.1 star (eg, 3.5 and 3.6), whereas the Apple store reported ratings with an accuracy of 0.5 star (eg, 3.5 and 4). In both stores, 5 stars was the best possible score. Of the 275 rated apps, the average rating was 3.84 stars. The number of raters per app ranged between 1 and 152,011, with an average of 2658 raters. However, 50 apps in the Apple store and 3 apps in the Android store did not have rating information available.

## Discussion

### Principal Findings

This review describes a comprehensive range of characteristics of available mobile solutions for MM, which help users understand and take their medication. An emphasis is placed on the functional features the current solutions offer and how this can inform future app development. Another main finding is related to the low transparency of information available in the app store description. We suggest that it is difficult for the user to identify the author’s affiliation and professional credentials, as reporting this information is not a standard in the app store descriptions.

### Market Overload—Difficult for Users to Navigate

When searching for MM apps, we found over 300 unique apps on the Apple and Android stores. A study by Martínez-Pérez examining mobile apps for the most prevalent health conditions reported that there were more than 1000 apps focusing on diabetes or depression [32]. This represents a large number of apps that the user must filter through to find the right one.

When one looks for an online solution for their health condition, well-established requirements for health interventions, such as providing evidence-based content and clinical effectiveness become pivotal, in addition to the qualities that are commonly sought out in any apps, such as user-friendliness and engagement [33,34].

### Low Indication of Author Affiliation

Another aspect of the health apps that would be helpful for the user to know during the process of choosing an app is the topic-related credibility of the author. However, during the solution assessment, we observed that the majority of apps did not mention the credentials of the author or professionals who helped codevelop the app in the app store description. Currently, the 2 main app stores require app authors to list their name (termed *seller* in the Apple store and *developer* in the Android store) and website if available. However, listing the qualifications or affiliations of the author is currently not a store requirement. Therefore, very few apps include this information in the app store description. Similarly, many other studies reviewing mobile apps for a variety of health conditions described that author affiliation and reporting of content sources in the app store description were infrequent [24,25,35,36]. The earlier Towards Evaluation and Certification of Telematics Services for Health project focusing on quality and safety in health informatics, recommends that the identity and qualification of the health care professional responsible for the clinical element of the software design should be one of the labeling requirements for clinical software.

We continued to look further for the authors’ credentials and conducted Google searches using the website and the name listed in the app store. We did so to find more information on the subject and examine the authors’ affiliations for the purpose of this study. However, as this is time consuming, we do not predict users would routinely do the same.

We found a lack of health care professional involvement (14.6%; 48/328) in app development. Furthermore, even fewer apps were codeveloped by academia. In addition, a few apps were authored by other organizations such as charities, nonprofits, government organizations, insurance companies, or pharmaceutical companies. Conversely, the majority of solutions were developed by software developers or tech companies. The previous literature widely indicates that there is a lack of involvement of health professionals or academics in the development of health apps [37-39].

### Features

One of the most prevalent features was symptom or vital tracker. It provides a unique record of health outcome details over time that can prove helpful to both users and their health care providers. We suggest that tracking symptoms may also be beneficial within a broader treatment approach, where medication is an integrated component of a comprehensive strategy (including, eg, diet, physical exercise, psychotherapy, or other, depending on the health issue). In such cases, tracked health outcomes provide insight into the effects and synergies of the interventions and may help optimize the treatment for each user. Future development could offer alternatives to automatically track data through wearables or new upcoming technologies, increasing and facilitating utilization of tracking features as a result.

There is an increasing number of apps (21.0%; 69/328) with the option to synchronize data from the MM app with a different app or device, including wearables (eg, Apple Watch). A similar review from 2013 did not report synchronization as a prevalent feature [15], which may be because of the focus of the previous study or market changes. We suggest that future development can further build on the synchronization efforts and reflect usage of wearables or other devices prevalent in society. This may improve the functionality of MM apps, including the tracking options described in the previous paragraph, as well as engagement of users in the treatment.

A majority of the reviewed apps offered a medication reminder. A study by Ahmed et al focusing on adherence apps has also described other adherence strategies such as gamification, in addition to the common reminder [21]. Another common feature was the option to share data with another person, for example, a doctor or a family member.

In addition to features helping users take their medication, almost one-fifth of the apps included an education component to help users understand the medication or the treatment process. Examples include information about correct usage, side effects, and interactions. For future development, we suggest also covering common patient concerns (legitimate vs myths) and desired treatment outcomes based on the evidence. The education section could be written in an easy-to-understand language and provide an added value compared with the patient information sheet, which is usually provided together with a prescription medication.

Some of the interesting novel features identified in this study were easier data entry for new medication (eg, barcode scanning and voice entry), correlations based on data entry, providing warnings if safe dosage exceeded, communication with health professional, and safety plan for acute or emergency situations. In addition, an increasing number of apps included in their tracking and journaling features options supporting healthy lifestyle choices such as physical activity; social interactions; food diary; awareness of emotions, mood, and thoughts; accomplishments; and triggers. Many of the novel features could inform future development, especially ones that make the use of mobile MM increasingly effortless, for example, easier data entry options, and features empowering the user, for example, individualized and improved tracking features.

### Other Characteristics

Although most of the apps were not specialized for MM of a particular drug or condition, there were several apps with a focus on a specific medical condition or specialty. The greatest number of apps were for MM of diabetes and apps helping women keep track of their reproductive health, including reminders for contraceptive pills.

When looking at the distribution of prevalent features by different specialties, diabetes apps had the greatest variety of features. When this fact is taken into consideration alongside the advancement in engagement and gamification [40,41], MM apps for diabetes can serve as a potential innovation model for other specialties. Although only a few apps focused on mental health MM, they were some of the most comprehensive ones in terms of features and content and had the highest prevalence of the education component.

### Initiatives for User Navigation

This study offers an overview of the app market for professionals in the field of eHealth and can inform development of new innovative solutions. On the other hand, the perspective of the user (the patient or the health consumer) remains crucial. For them, a highly dynamic approach including up-to-date transparent overviews of characteristics and evaluations of solutions would be very beneficial and would empower them to *understand and manage* their medication with the support of a mobile app. The pivotal first step for the user is to select an appropriate app.

There are several initiatives aiming to help users navigate the app market, such as Apps Library by the National Health Library in the United Kingdom, RankedHealth or Psyberguide [42-44]. Health On The Net Foundation provides a certification (HONcode) to health-related websites providing reliable health information and meeting the standards for ethics and transparency [45,46]. The Ontario Telemedicine Network has launched a website called Practical Apps, which publishes reviews on specific health topics and can be used by public or health care providers recommending apps to their patients [47]. Previous literature states that developing such an initiative remains a challenge with little success thus far [24,48]. Deshpande and Jadad suggest a *crowd-sourcing collaborative approach*, similar to Wikipedia, as a potential solution [24,48].

A future direction could be an online registry and review aggregation website, similar to Metacritic, which is used for film and other media. The future platform could contain structured overviews of various characteristics, reviews by professionals (trained health care *critics*) including an *evidence-based score*, and reviews by users, all in one place.

The professional reviews could follow some of the standardized frameworks for quality assessment of mobile apps, for example, Mobile App Rating Scale [49], suitable for a wide range of health apps; a recently published assessment framework specifically for mental health apps [50]; or other published frameworks [33,51-54].

### Limitations

One of the potential limitations of this review is using the information available in the app store description, without downloading and fully testing the apps out. The apps themselves potentially may have additional functionalities not described in the app store description and that may have impacted the results presented in this study. However, this way of reviewing the current apps and collecting their characteristics mimics the experience of the user when they are deciding whether to download or purchase an app or not, and thus, it bridges the gap between reality and research. This is of particular value because the fact whether an app will be actually used or just be in the app store with minimal downloads is based in the first place on the information available in the app store description. Developers are aware of this and strongly motivated to list their apps’ features. If a major feature is not mentioned in the app store description and visible at first sight, usually the app naturally gets passed over. In addition, the proposed study focused on collecting the objective characteristics of the apps and did not aim to do a qualitative evaluation of the solutions.

A second limitation is related to the time difference between data collection (January 2017) and time of submission of the publication, considering the fast-evolving landscape of the app market. Moreover, there was also a 1-year time difference between the search and categorization. The disadvantage of this is that 34 apps were discontinued during this period. The advantage might be that the rest of the apps (328) that were categorized, and their characteristics are outlined in this study, are the sustained ones that remained available over a year. Third, identification of app duplicates was limited to the duplicates within one app store. As store requirements are different between the Android and Apple store, we were not able to reliably identify duplicates across 2 different platforms.

The inclusion criteria included English language. Therefore, the findings are not representative of all MM apps available in the global market offered in languages other than English. Moreover, both researchers were physically present in Canada during the search process. Even though substantial effort was put into increasing the anonymity during the search, a search in another country may have returned different results. Similarly, despite these efforts, the search results may also be affected by the app stores’ algorithms. Specifically, the ranking of the results may be affected by various factors, including but not limited to how many people have clicked on the app title before, the user rating, increase of downloads in the past month, or the number of downloads per day.

### Conclusions

Mobile apps have the potential to empower patients with personalized immediate support and improve compliance with the treatment and engagement in long-term well-being. However, searching for an appropriate MM app is challenging because the marketplace offers hundreds of solutions which makes it difficult for the user to filter through. To find a trustworthy app, it would be beneficial for the user to have the information about the content source or author affiliation easily available during the search. However, this study found a lack of reporting of the author affiliation in app store descriptions. Therefore, the study highlights the need for improved standards for reporting on app stores. In addition, it underlines the need for a platform to offer users of health apps an ongoing evaluation by health professionals in addition to other users. The aim of such efforts would be to provide users with tools to readily assess the credibility of eHealth solutions before making their choice.

The study reports that prevalent features of available MM apps were reminder, symptom tracker, and ability to share data with a family member or doctor. The results presented in the study help inform the theoretical and practical approach for app development, in particular, decisions related to the content selection process. Building on the existing experience, it is important to start working on the next generation of solutions, which will improve engagement of users in the treatment process. Future development may include improved tracking features to optimize treatment for each user based on their exact treatment outcome data, as well as components making the use of mobile MM increasingly effortless (eg, easier data entry options). Moreover, emphasis could be put on a broader comprehensive treatment approach framing medication as its integrated component instead of a stand-alone intervention.

## Abbreviations

eHealth: electronic health
MM: medication management
